# Supplementary figures and images for: Replication stress and FOXM1 drive radiation induced genomic instability and cell transformation
Source: PLoS One. 2020 Nov 30;15(11):e0235998. doi: 10.1371/journal.pone.0235998 (PMC7703902; doi:10.1371/journal.pone.0235998)

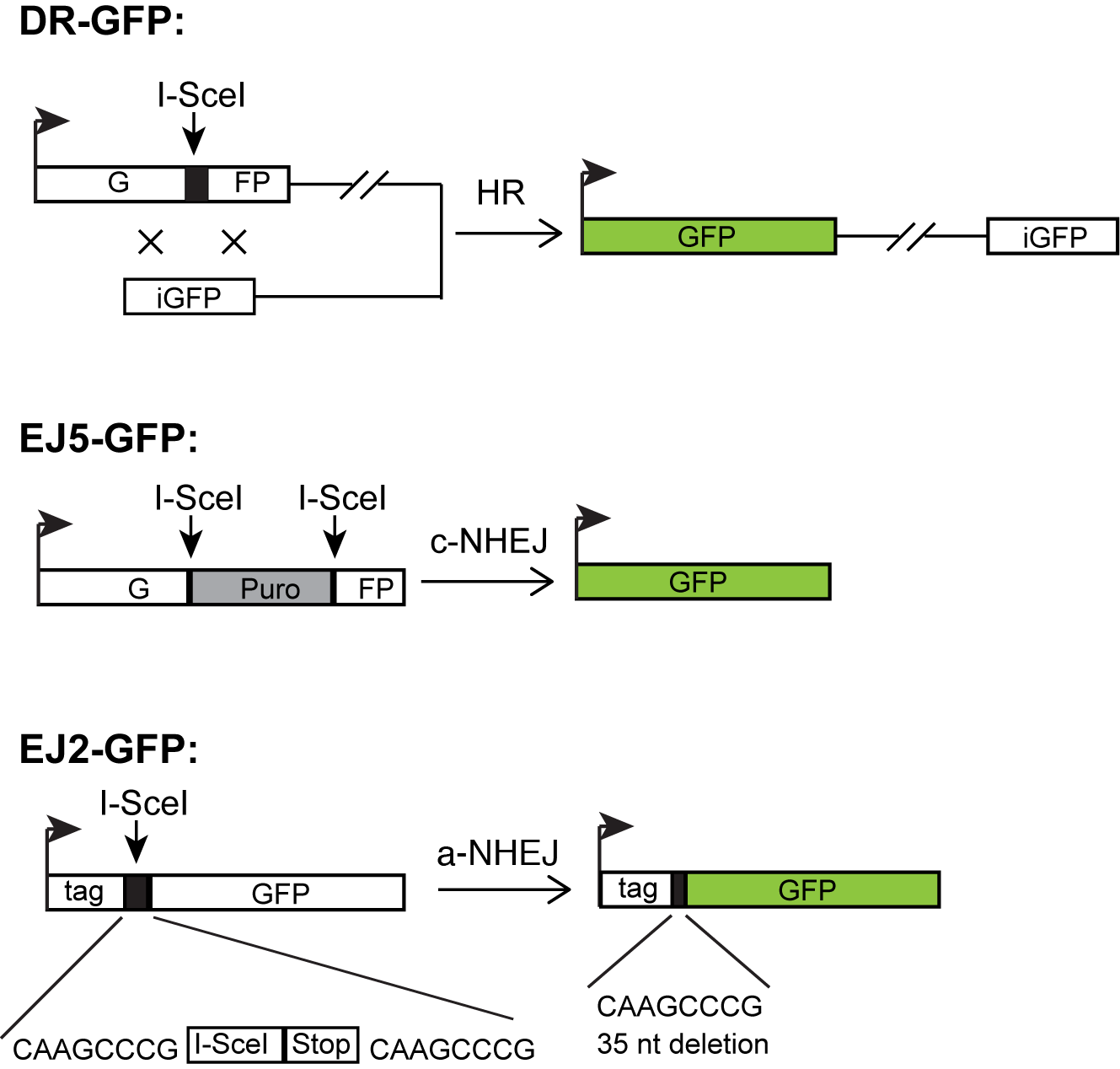

Supplement: S1 Fig — GFP expression is gained when repair occurs by the specific mechanism. (TIF) [file pone.0235998.s001.tif]

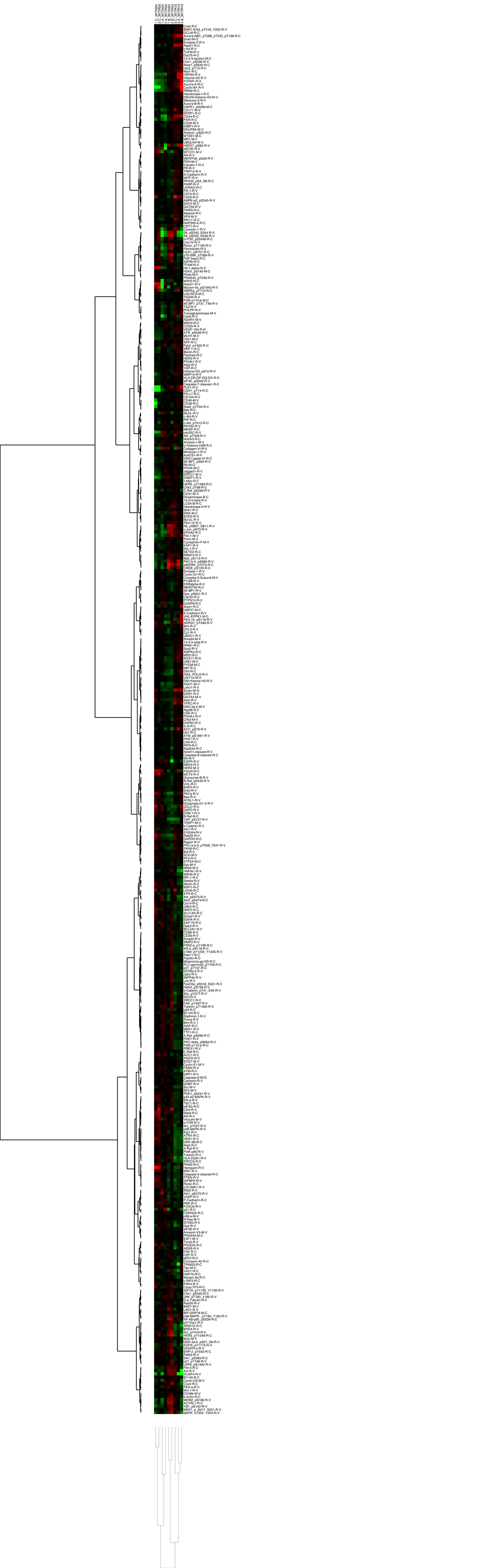

Supplement: S2 Fig — Triplicate samples of non-irradiated (NI) or 2Gy irradiated HBEC3-KT cell lysates collected at day 5 or day 7. Heat map represents ‘‘rank-ordered” changes induced by each treatment, calculated by summing median-centered normalized protein amount. (TIF) [file pone.0235998.s002.tif]

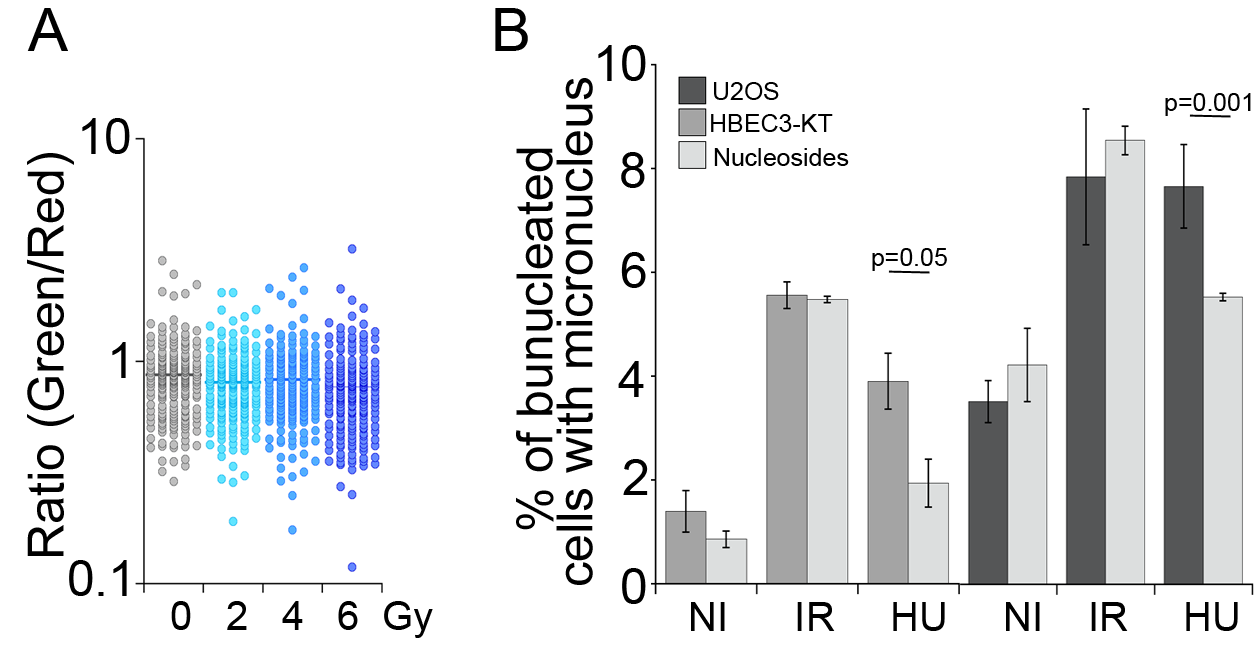

Supplement: S3 Fig — A) Asymmetry of replication track: the graph depicts the ratio of CldU/IdU track length for each dose. No statistical divergence following One Way ANOVA analysis. B) Micronucleus formation rates in HBEC3-KT or U2OS cells were irradiated or treated for 48h with 25μM HU with or without 30μM nucleosides. Error bars are SEM. Student’ t-test. (TIF) [file pone.0235998.s003.tif]

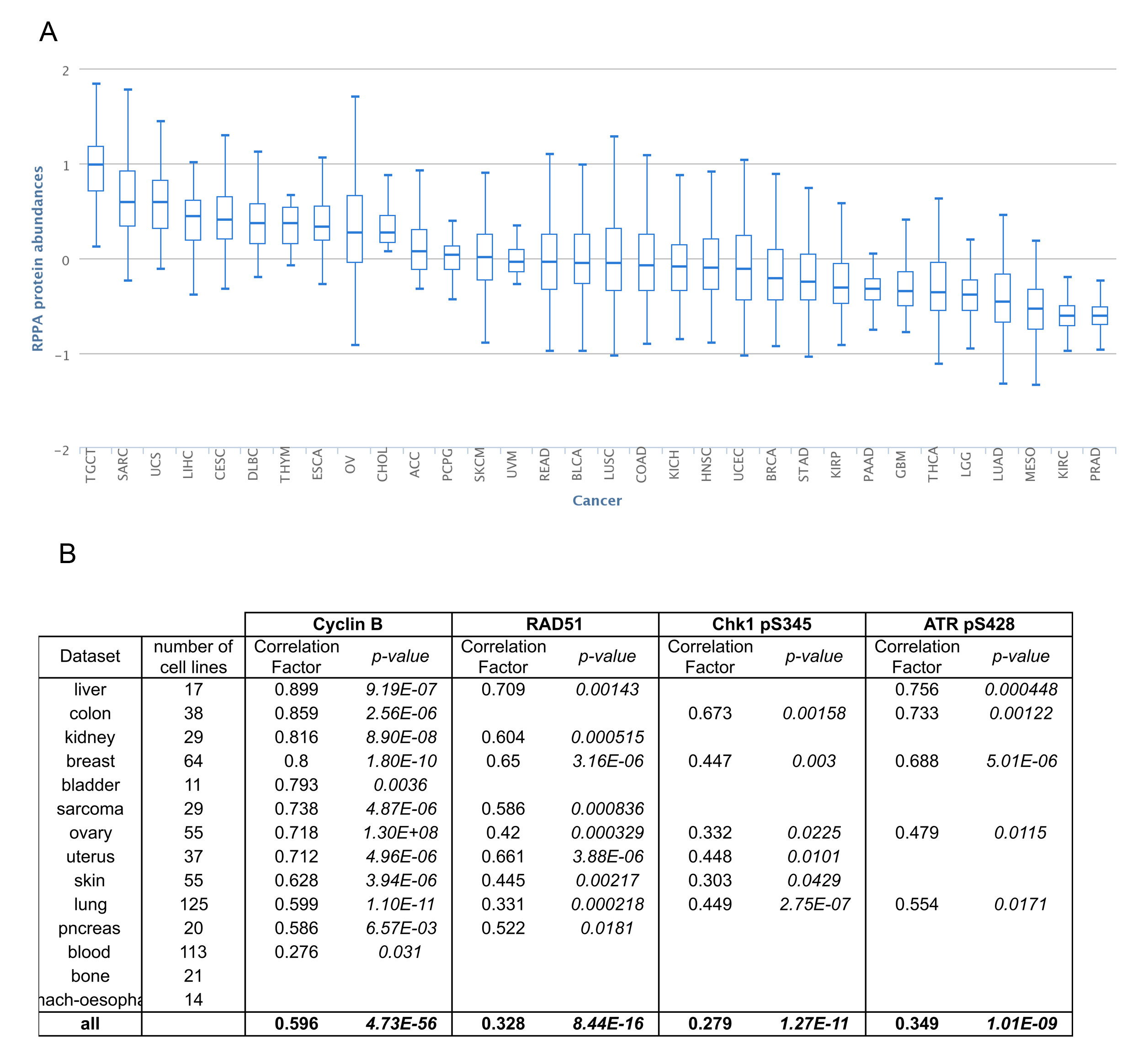

Supplement: S4 Fig — A) Relative FOXM1 expression across datasets of cell lines grouped by cancer type extracted from the MD Anderson Cell Lines Project Portal. https://tcpaportal.org/mclp/#/ B) Table listing the correlation factor and significance of paired comparison of the indicated protein with FOXM1 expression in each dataset of cell lines grouped by cancer types. Included are the comparisons that were significant (p≤ 0.05). (TIF) [file pone.0235998.s004.tif]

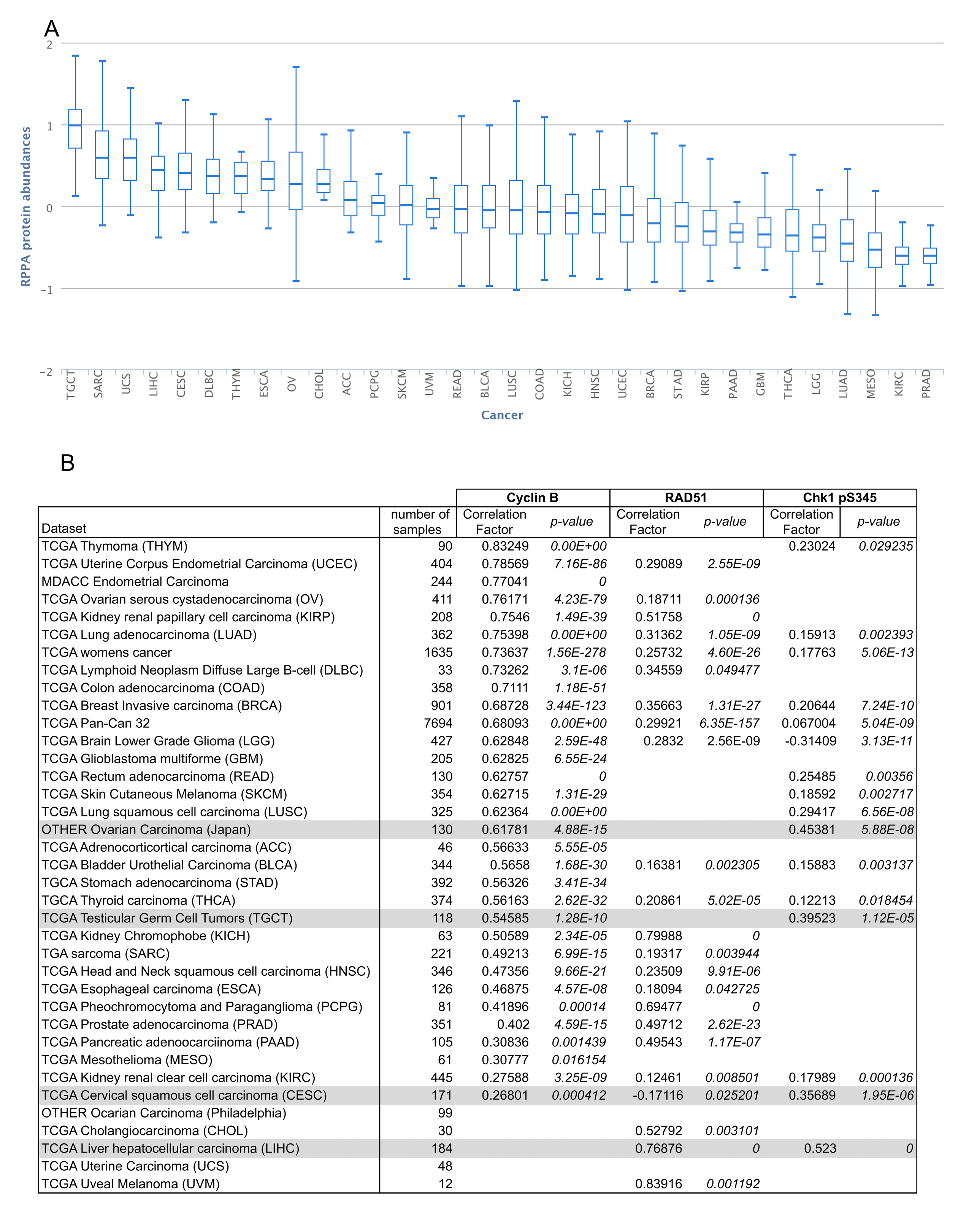

Supplement: S5 Fig — A) Relative FOXM1 expression across datasets of tumor samples grouped by cancer type extracted from the The Cancer Proteome Atlas. https://tcpaportal.org/tcpa/. B) Table listing the correlation factor and significance of paired comparison of the indicated protein with FOXM1 expression in each dataset of tumor samples grouped by cancer types. Included are the comparisons that were significant (p≤ 0.05). (TIF) [file pone.0235998.s005.tif]

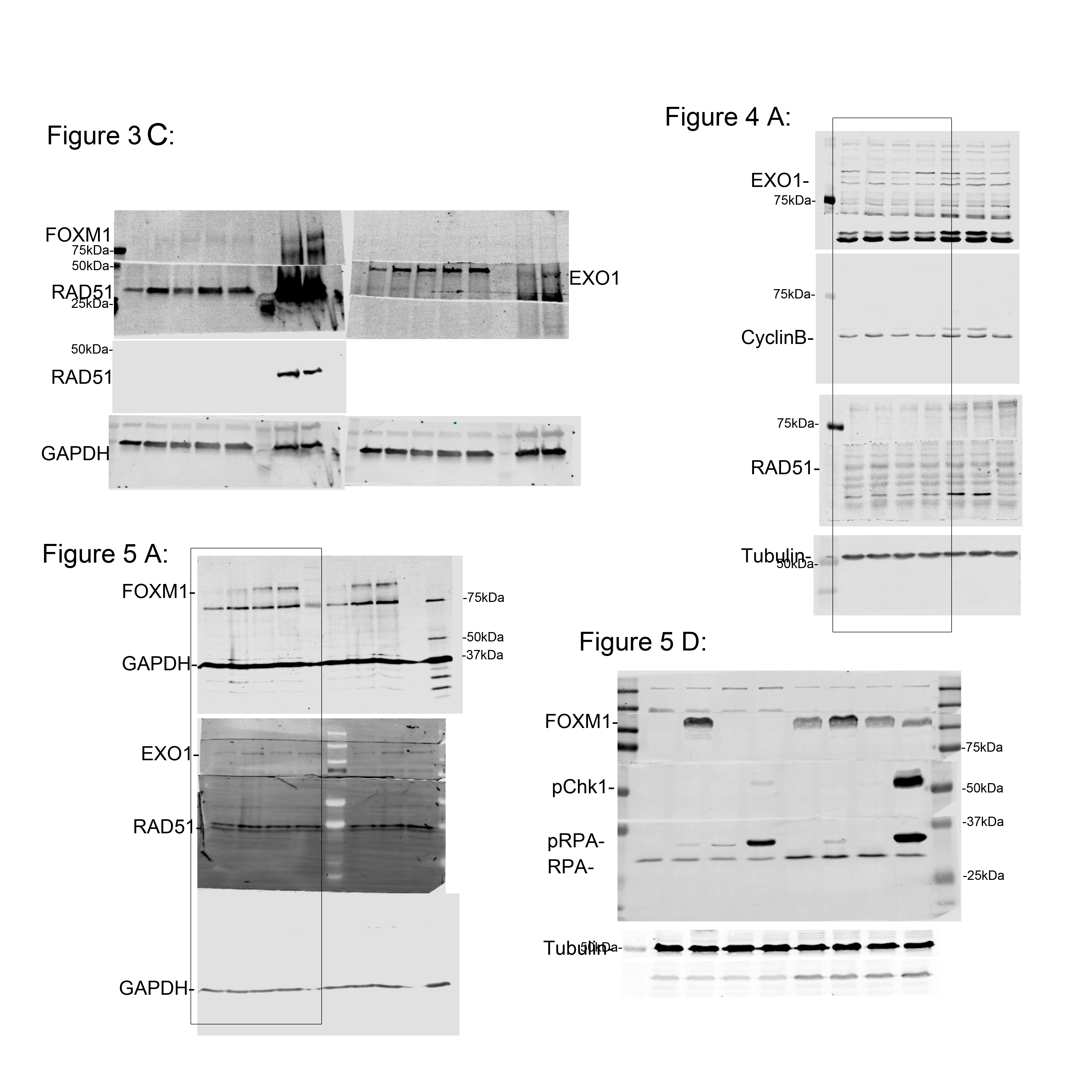

Supplement: S1 Raw images — (TIF) [file pone.0235998.s006.tif]
